# Supplementary material for: Chromatin interactome mapping at 139 independent breast cancer risk signals
Source: Genome Biol. 2020 Jan 7;21:8. doi: 10.1186/s13059-019-1877-y (PMC6947858; doi:10.1186/s13059-019-1877-y)
Supplement: Supplementary file 1 — Additional file 1. Supplementary figures. [file 13059_2019_1877_MOESM1_ESM.docx]

**Supplementary Figures**

Fig. S1


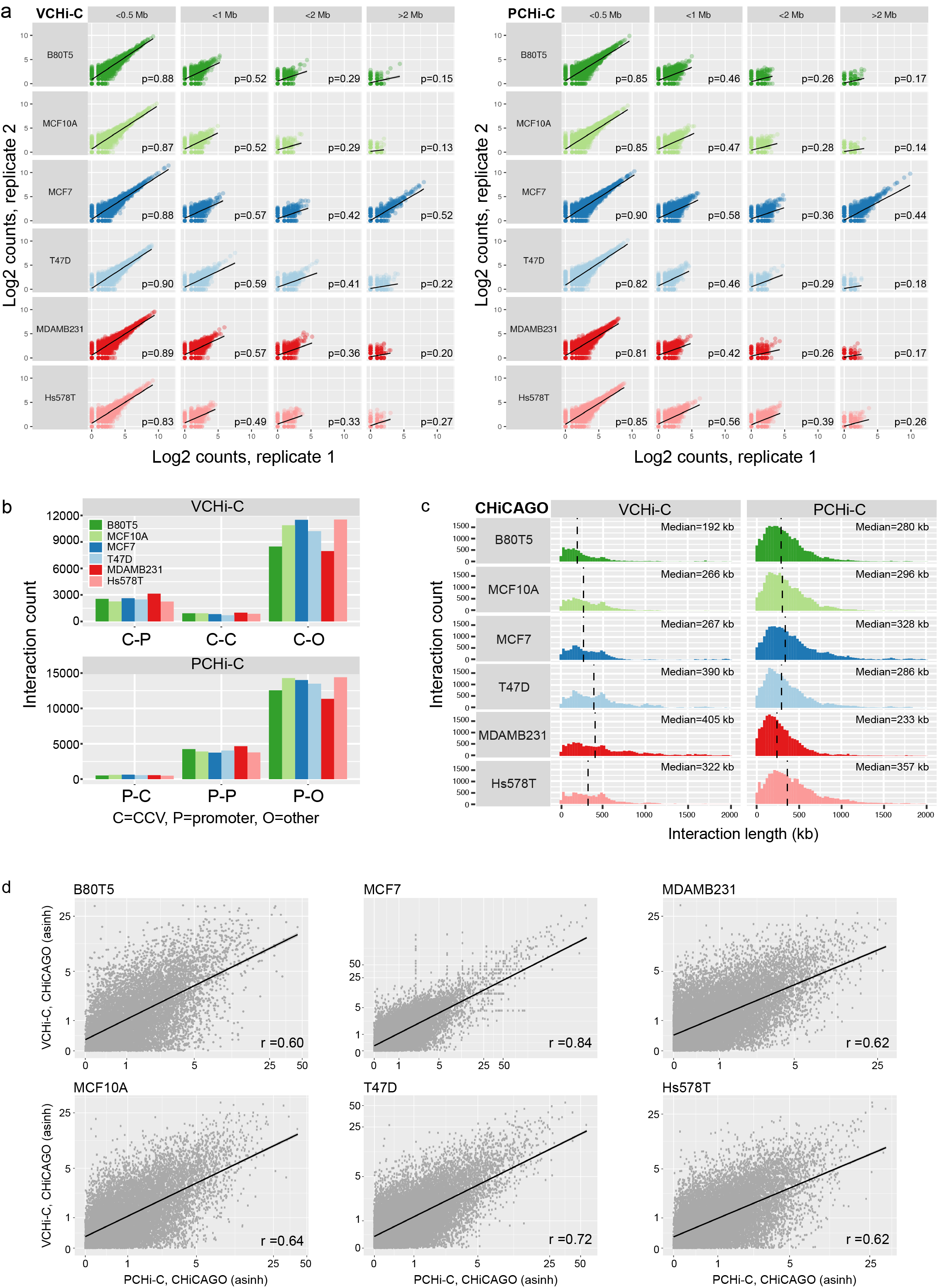


**VCHi-C and PCHi-C CHiCAGO-identified interaction characteristics.** **a** Scatter plots showing the correlation between duplicate VCHi-C or PCHi-C libraries based on the number of raw di-tags mapping to interaction fragment pairs. The analysis was stratified by cell line (rows) and distance between interacting fragments (columns). ρ is Spearman’s correlation; the black lines represent the linear regression fit. **b** The abundance of different classes of CHiCAGO-scored VCHi-C (upper panel) and PCHi-C (lower panel) interactions. **c** Distribution of CHiCAGO-scored interaction lengths in each breast cell line. Dashed black vertical lines denote the median interaction length. **d** Scatter plots showing the concordance of inverse hyperbolic sine (asinh)-transformed CHiCAGO-scored VCHi-C versus PCHi-C interactions in the respective breast cell lines. r is Pearson’s correlation; the black lines represent the linear regression fit.

Fig. S2


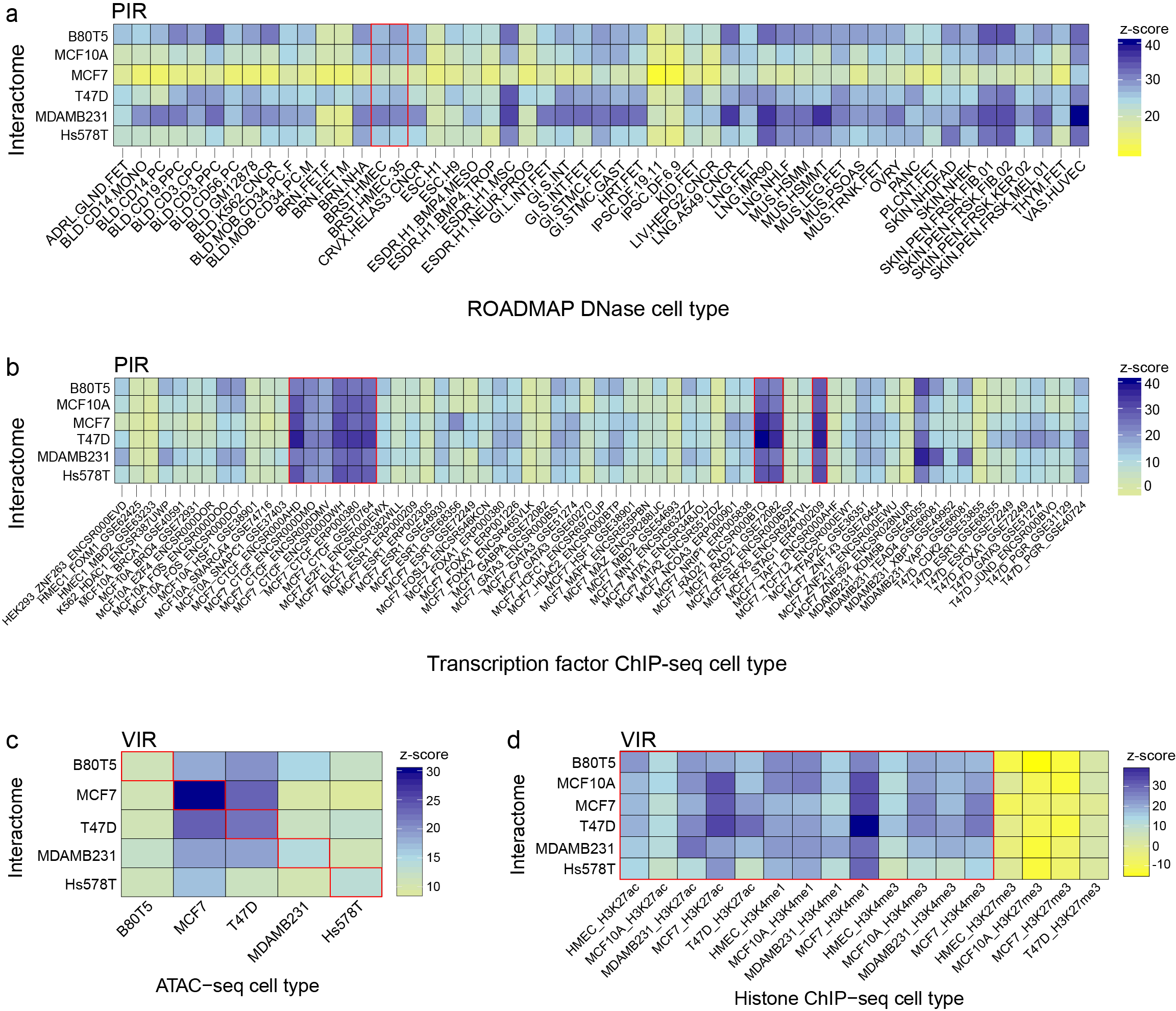


**Promoter-interacting regions (PIRs) and variant-interacting regions (VIRs) are enriched for breast-specific regulatory features.** Heatmaps showing PIR enrichment for **a** DNase I hypersensitivity sites in a ROADMAP cell types, **b** transcription factor binding in breast (additional datasets) and other cell types, expressed as z-scores. Heatmaps showing VIR enrichment for **c** ATAC-seq peaks in breast cell lines and **d** histone marks by ChIP-seq in available breast cell lines, expressed as z-scores. The red outlines highlight key enrichment signals.

Fig. S3


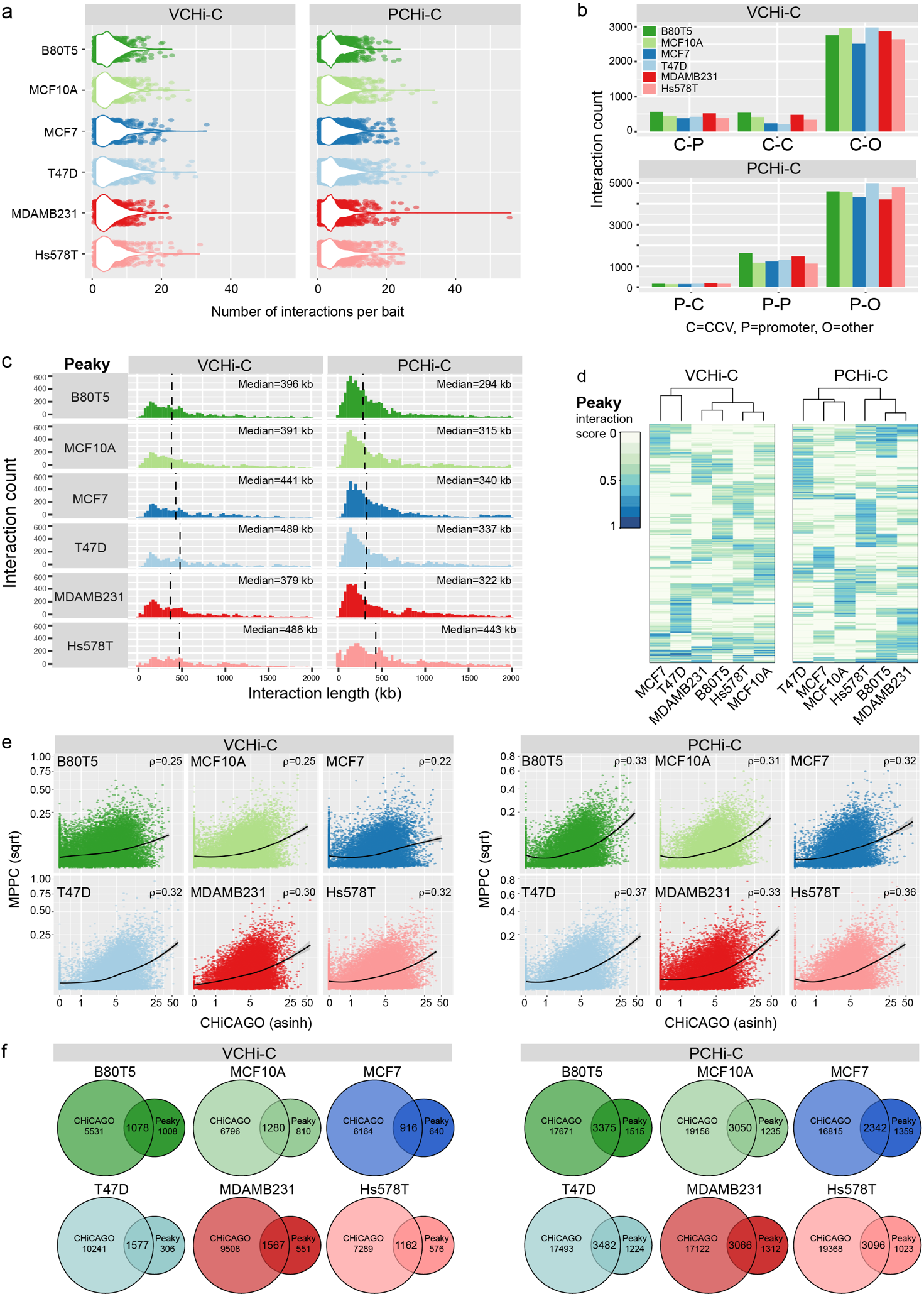


**VCHi-C and PCHi-C Peaky-identified interaction characteristics.** **a** Distribution of Peaky-scored interaction number per bait per cell line (combined biological replicates). **b** The abundance of different classes of Peaky-scored VCHi-C (upper panel) and PCHi-C (lower panel) interactions. **c** Distribution of Peaky-scored interaction lengths in each breast cell line. Dashed black vertical lines denote the median interaction length. **d** Agglomerative hierarchical clustering for the VCHi-C and PCHi-C. **e** Scatter plots showing the correlation between CHiCAGO (inverse hyperbolic sine-transformed) and Peaky (square root-transformed) interaction scores. ρ is Spearman’s correlation; the black lines represent the loess smoothed fit. **f** Venn diagrams illustrating the overlap in CHiCAGO- and Peaky-scored interactions in each capture per cell line.

Fig. S4


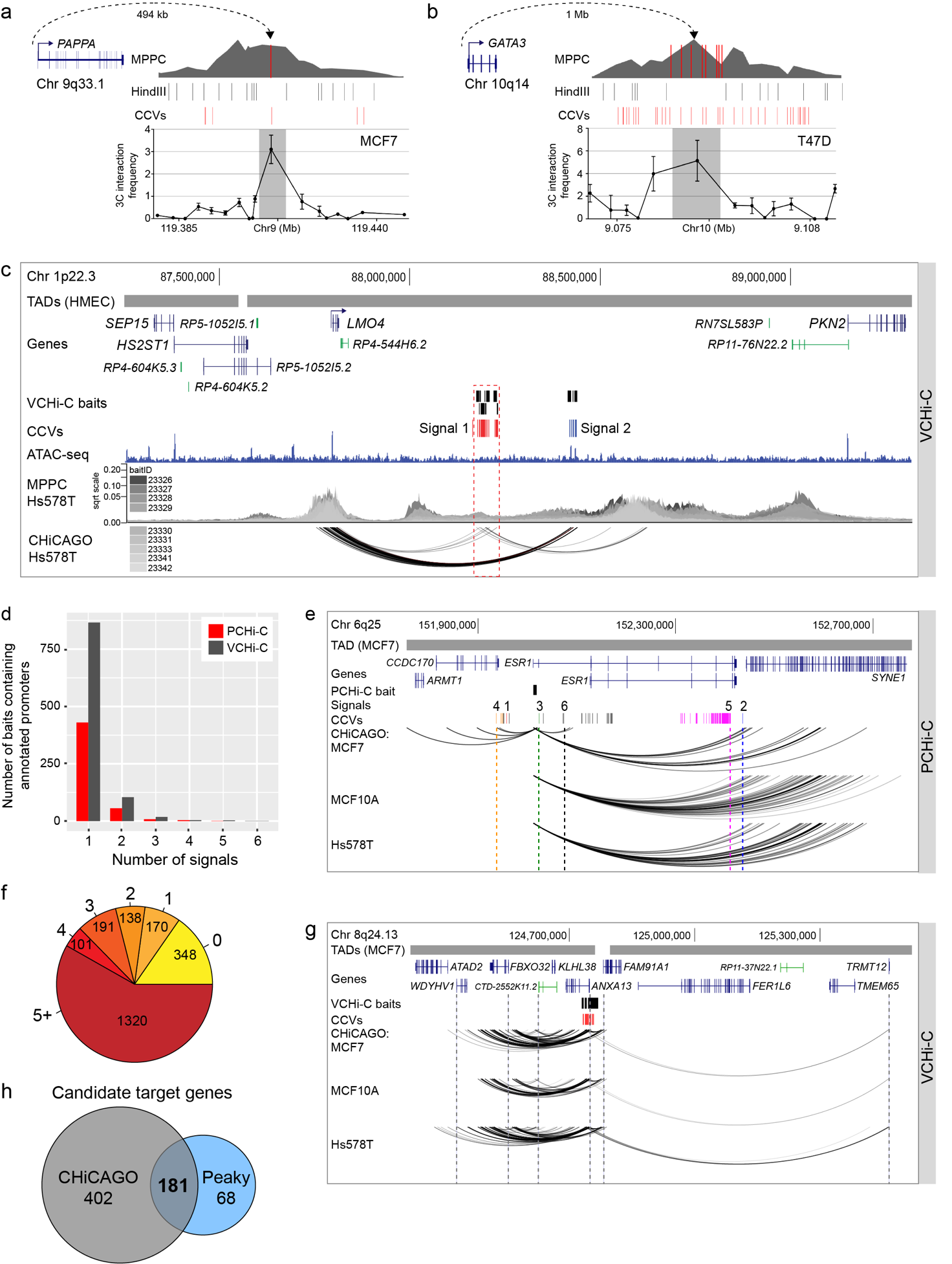


**Candidate target gene properties. a** 3C interaction profiles at 9q33.1 between the *PAPPA* promoter and CCVs in MCF7 cells. The anchor point is set at the *PAPPA* promoter. Error bars represent SD (n = 3). **b** 3C interaction profiles at 10q14 between the *GATA3* promoter and CCVs in T47D cells. The anchor point is set at the *GATA3* promoter. Error bars represent SD (n = 3). **c** Chromatin interactions at 1p22.3 in Hs578T cells. Topologically associating domains (TADs) are shown as horizontal gray bars above GENCODE annotated coding (blue) and non-coding (green) genes. The VCHi-C baits are depicted as black boxes. Risk signals 1 and 2 are numbered and the CCVs within each signal are shown as colored vertical lines. The ATAC-seq track is shown as a dark blue histogram. Peaky defined MPPC values (from nine specified BaitIDs) are plotted. CHiCAGO-scored interactions are shown as black arcs. The dashed red outline highlights the signal 1 CCVs. **d** Correlation between the number of candidate target genes and independent risk signals in the PCHi-C and VCHi-C datasets. **e** Chromatin interactions at 6q25 in MCF7, MCF10A and Hs578T breast cell lines. Topologically associating domains (TADs) are shown as horizontal gray bars above GENCODE annotated coding (blue) genes. The *ESR1* PCHi-C bait (BaitID: 355261) is depicted as a black box. Risk signals 1-6 are numbered and the CCVs within each signal are shown as colored vertical lines. CHiCAGO-scored interactions are shown as black arcs. The dashed colored vertical lines highlight *ESR1* promoter-signal interactions. **f** Enumeration of the number of transcription start sites skipped during chromatin looping for the 651 target gene promoter interactions. **g** Chromatin interactions at 8q24.13 in MCF7, MCF10A and Hs578T breast cell lines. Topologically associating domains (TADs) are shown as horizontal gray bars above GENCODE annotated coding (blue) and non-coding (green) genes. VCHi-C baits are depicted as black boxes and the signal 1 CCVs as red vertical lines. CHiCAGO-scored interactions are shown as black arcs. The dashed gray vertical lines highlight promoter-signal interactions to the candidate target genes. **h** Venn diagram illustrating the number of candidate target genes identified by CHiCAGO, Peaky and both algorithms.

Fig. S5


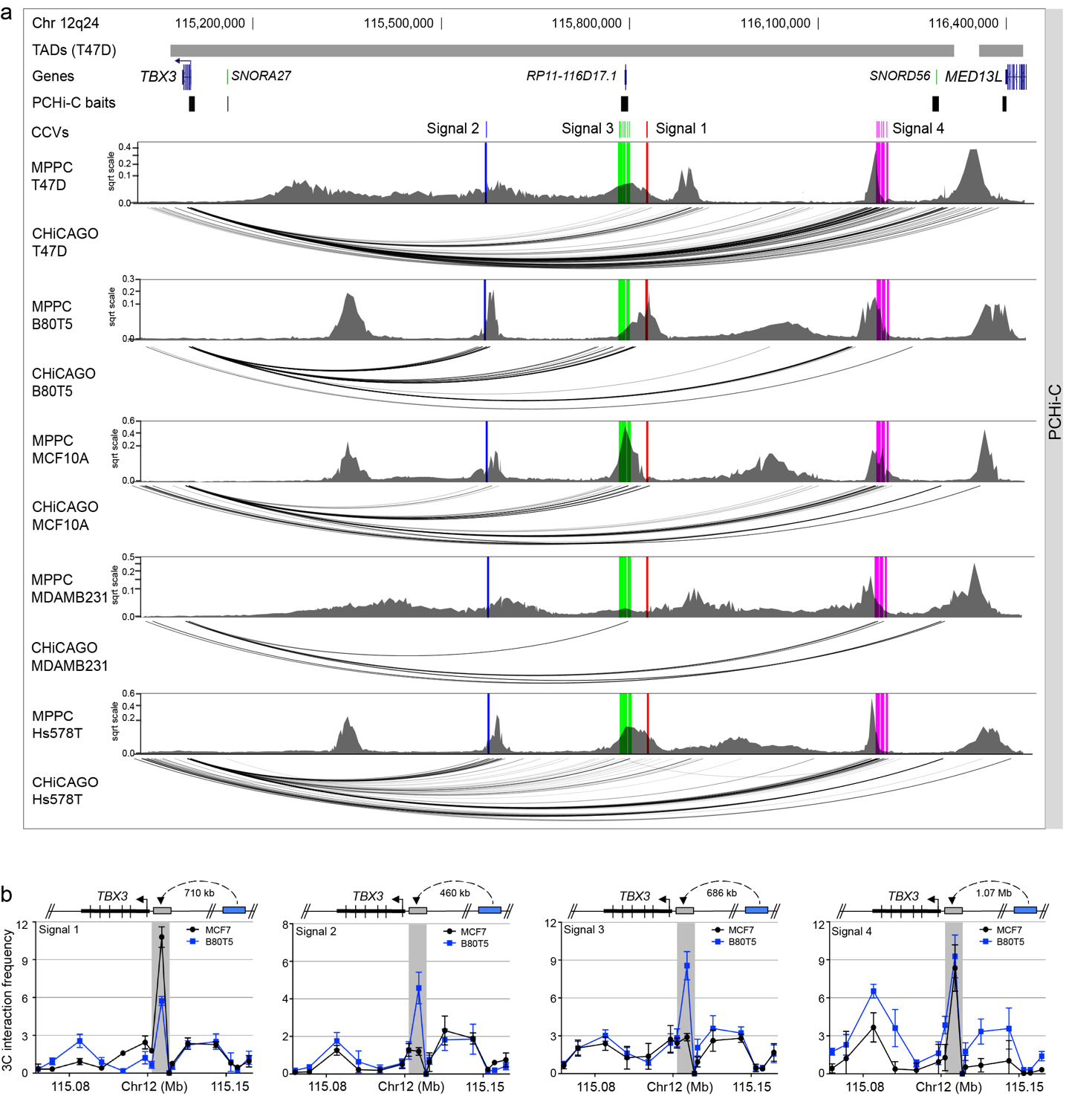


**Chromatin interactions across 12q24.** **a** Chromatin interactions at 12q24 in ER+ T47D, ER- MDAMB231 and Hs578T breast cancer cell lines, and non-tumorigenic B80T5 and MCF10A breast cells. Topologically associating domains (TADs) are shown as horizontal gray bars above GENCODE annotated coding (blue) and non-coding (green) genes. PCHi-C baits are depicted as black boxes. Risk signals 1-4 are numbered and the CCVs within each signal are shown as colored vertical lines. Peaky defined MPPC values (from PCHi-C baitID 596031) are plotted with the CCVs overlaid as colored vertical lines. CHiCAGO-scored interactions are shown as black arcs. **b** 3C interaction profiles between the risk signals 1-4 and the *TBX3* promoter in MCF7 and B80T5 cell lines. Anchor points are set at signals 1-4. Error bars represent SD (n = 3).

Fig. S6


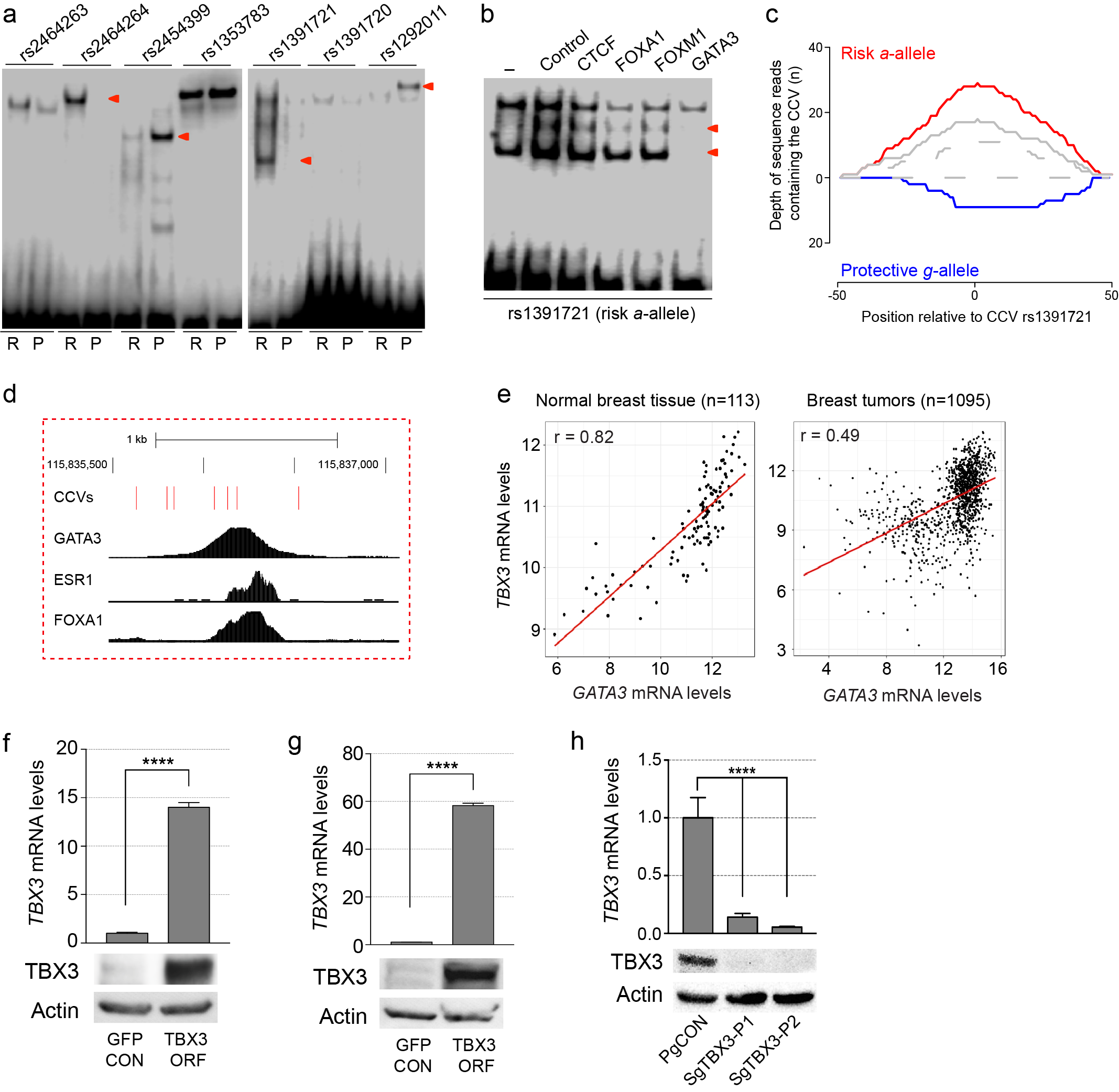


**Additional *in vitro* and *in vivo* studies for 12q24. a** EMSAs for signal 1 CCVs to detect allele-specific binding of nuclear proteins. Labeled oligonucleotide duplexes were incubated with BT474 nuclear extract. Red arrowheads show bands of different mobility detected between risk (R) and protective (P) alleles. **b** EMSAs for CCV rs1391721 to identify candidate nuclear proteins. Unlabeled competitor oligonucleotide duplexes for predicted transcription factors (100-fold molar excess) were incubated with labeled rs1391721-containing oligonucleotide duplex and MCF7 nuclear extract. Red arrowheads indicate bands that were competed for complex formation on the risk (*a*) allele. **c** Allele-specific GATA3 binding at CCV rs1391721 in heterozygous MCF7 cells. The depth of reads containing the risk (red) and protective (blue) alleles are shown. **d** Zoomed in view of Signal 1 CCVs at 12q24. CCVs are shown as red vertical lines. GATA3, ESR1 and FOXA1 binding are shown as black histograms. **e** Scatter plots of *TBX3* versus *GATA3* gene expression in TCGA normal breast tissue (n = 113, r is Pearson’s correlation) and breast tumors (n = 1095, r is Pearson’s correlation). **f** Top: *TBX3* levels in HMLE-control (GFP CON) and HMLE-TBX3 overexpressing (TBX3 ORF) cells assessed by qPCR and normalized to *GUSB*. Error bars represent SEM (n=3). Bottom: Western blot analysis of TBX3 and Actin, serving as a loading control, in matched cell samples. **g** Top: *TBX3* levels in MCF7-control (GFP CON) and MCF7-TBX3 overexpressing (TBX3 ORF) cells assessed by qPCR and normalized to *GUSB*. Error bars represent SEM (n = 3). Bottom: Western blot analysis of TBX3 and Actin in matched cell samples. **h** Top: TBX3 levels in MCF7-control (PgCON) and MCF7-TBX3-dCas9-KRAB repressed cells (SgTBX3-P1/P2) assessed by qPCR and normalized to *GUSB*. Error bars represent SEM (n = 3). Bottom: Western blot analysis of TBX3 and Actin in matched cell samples. **f-h** *P*-values were determined by a two-tailed *t*-test (****p < 0.0001).
